# Creep Age Forming of Fiber Metal Laminates: Effects of Process Time and Temperature and Stacking Sequence of Core Material

**DOI:** 10.3390/ma14247881

**Published:** 2021-12-20

**Authors:** Mehdi Safari, Ricardo Alves de Sousa, Fábio Fernandes, Mazaher Salamat-Talab, Arash Abdollahzadeh

**Affiliations:** 1 Department of Mechanical Engineering, Arak University of Technology, Arak 38181-8411, Iran; salamattalab@arakut.ac.ir (M.S.-T.); arash_abd@yahoo.com (A.A.); 2 Center for Mechanical Technology an Automation, Department of Mechanical Engineering, Campus de Santiago, University of Aveiro, 3810-183 Aveiro, Portugal; rsousa@ua.pt (R.A.d.S.); fabiofernandes@ua.pt (F.F.)

**Keywords:** creep age forming, fiber metal laminate, time, temperature, layout of fibers

## Abstract

Fiber metal laminates (FMLs) are a type of hybrid materials interlacing composites and metals. In the present work, FMLs with aluminum alloy 6061 as the skin and E-glass fiber-reinforced polypropylene (PP) as the core material are fabricated and formed by the creep age forming (CAF) process. The effects of time and temperature as the process parameters and thickness and stacking sequences of composites layers as the FML parameters are evaluated on the springback of glass-reinforced aluminum laminates (GLARE) FMLs. After the CAF process, the springback of creep age-formed FMLs is calculated. The results show that the FMLs can be successfully formed with the CAF process by considering appropriate time and temperature. In addition, the stacking sequence of composite layers can affect the springback behavior of FMLs significantly.

## 1. Introduction

Fiber metal laminates (FMLs) are hybrid materials interlacing composites and metals. The high strength to weight ratio of FMLs makes these composites desirable for use as an ideal lightweight structural material. Since aluminum is the most widely used alloy in the aircraft industry, aluminum-based FMLs have been further developed. There are three kinds of FMLs: aramid-reinforced aluminum laminates (ARALL), carbon-reinforced aluminum laminates (CARALL) and glass-reinforced aluminum laminates (GLARE) [1]. GLARE is a well-known type of FML that has numerous industrial applications. In recent years, some developments have been made regarding FMLs. Heggemann and Homberg [2] studied the deep drawing process of FMLs. They concluded that by implementing the pre-curing processes before the deep drawing process, the dimensional accuracy could be considerably improved. Blala et al. [3], using variable blank holder force, improved the drawability of FMLs in fabrication of cylindrical surfaces with the deep drawing process. Saadatfard et al. [4] studied the drawability of FMLs in the hydromechanical drawing (HMD) process, concluding that necking happens at the lower metal sheet near the pole of the drawn part. Hahn et al. [5] predicted the forces in the stamping process of FMLs at thermoforming temperature with an analytical model. Rahiminejad and Compston [6] investigated the effect of pre-heat temperature on formability of steel-based FMLs in stretch forming. They concluded that a pre-heat temperature of 140 °C improves the formability of FML. For stamp forming processes of FMLs, Blala et al. [7] showed that the blank holder force and blank holder gap can improve the quality and forming depth. Li et al. [8] investigated the shot peen forming of FMLs based on aluminum-lithium alloy. Their results showed that the shot-peened layer withstands the plastic deformations while elastic deformations are applied in the other layers. Zhang et al. [9] predicted the deformed shape of FMLs after laser peen forming, using eigenstrain-based modeling in order to obtain the eigenstrains induced in metal layers of FML. Gisario and Barletta [10] investigated the laser forming process of GLARE and found that an increasing number of irradiating paths decreases the bending angle. In another research study, Gisario et al. [11] proposed a prediction model based on neural network models for laser forming of FMLs to estimate temperature and bending angle. Blala et al. [12] investigated the effect of blank holder gap (BHG) in the hydroforming process of FMLs. They concluded that the BHG smaller than the initial thickness of FML can improve the quality and depth of forming of FML hydroformed specimens. Chernikov et al. [13] compared the electromagnetic formed FMLs with the rubber pad forming process and proved that the formability was increased in the electromagnetic forming process. In a parametric study, Werner et al. [14] concluded that the tension–compression behavior of the fibers has an important effect in the forming of FMLs. Still regarding the formability of the FMLs, Mennecart et al. [15] studied the effects of fibers and concluded that because of contacting the fibers with metal blanks, the formability of FMLs will be reduced. Liu et al. [16] studied the effect of fiber orientation on the formability of FMLs and concluded that the unidirectional and multidirectional fiber in the middle layer has important effects on the thinning of FMLs. Jalali Aghchai and Khatami [17] studied the effect of skin and core thickness on the formability of FML and showed that the average formability increases with doubling of core and skin thickness. Logesh and Raja [18] studied the mechanical properties and formability of the FMLs by adding the Mg–Al layered double hydroxide (LDH) and showed that the FMLs with the Mg–Al LDH had better formability compared to FMLs without LDH.

It should be noted that with stress relaxation after the forming process, large amounts of springback may occur, decreasing the dimensional accuracy of the formed specimens. Therefore, the study of springback is vital. Keipour and Gerdooei [19] studied the effect of lay-up on the springback of FML and proved that the asymmetric lay-up for fibers with longitudinal direction influenced the springback behavior of FML while the core thickness had a little effect. Isiktas et al. [20] investigated the springback in the V-bending process of FMLs with carbon fiber-reinforced cores and concluded that the springback in the formed FML was increased by increasing the bending angle and thickness, and also decreasing the core thickness. Zal et al. [21] studied the effects of temperature and lay-up on spring back of FMLs and concluded that 160 °C was the minimum proper forming temperature with [45/−45] and [0/90] lay-ups.

Over recent decades, forming of aluminum airfoils with usual forming methods has been limited. To overcome this limitation, Textron Aero structures [22] suggested a recent forming method with the ability to form the complex shapes with acceptable strength. This proposed forming method is called creep age forming (CAF) and is basically related to the release and/or creep with the artificial aging of a metal alloy. The CAF contains three steps, as follows: (1) Loading the plate to fit in the die; (2) Heating the die and plate at a specific time and temperature in such a way that hardening and stress release occurs, simultaneously strengthening due to precipitation, and forming the plate is done; (3) Unloading. In the following, some reports on the fabrication of specimens by CAF process with a focus on springback in the CAFed specimens are presented. Li et al. [23] proposed an accelerated method for compensating of the springback in CAF. They concluded that the proposed accelerated method can considerably improve the efficiency of tool design in order to decrease the springback of CAFed specimens. In another work, Li et al. [24] studied the effect of residual stresses induced during the machining process on the springback of CAFed aluminum specimens in asymmetric creep forming and showed that the prediction of springback can considerably improve by considering the effects of residual stresses. Also, Li et al. [25] compared the springback behavior for asymmetric and symmetric CAF processes for an Al–Cu–Li alloy (AA2050). They concluded that the asymmetric models can better predict the springback behavior of CAFed specimens. Shahverdi and Alipour Mogadam [26] studied the effects of time and temperature on springback of CAFed aluminum alloy 7075. They showed that the springback of AA7075 decreases by increasing time and temperature. Safari et al. [27] studied the statistical modeling, sensitivity analysis and multi-objective optimization of CAF of aluminum AA7075 tailor-machined blanks (TMB). They investigated the effects of time and temperature on springback of TMBs after the CAF process. Their results showed that, in the range of considered times and temperatures, the springback of thin and thick sections of TMB is decreased by increasing the time and temperature. Safari et al. [28] was focused on the effect of process parameters, i.e., time and temperature, on the springback of the GLARE. In addition, two types of the composite materials were used in core of the specimens, i.e., E-glass/pp and E-glass/PA6. Their results reveals that the core material has a significant effect on the springback of the GLARE specimens.

Based on the authors’ knowledge, no research has so far been presented on the simultaneous study of the effects of process and FML parameters on the springback for creep age formed FMLs. Hence, in this paper, the CAF of FMLs is experimentally studied, and for this purpose, the effect of stacking sequences of composites layer in core material and thickness of the core material, as well as process temperature and time on the springback of the samples were investigated.

## 2. Materials and Methods

### 2.1. FML Preparation

**Figure 1:**
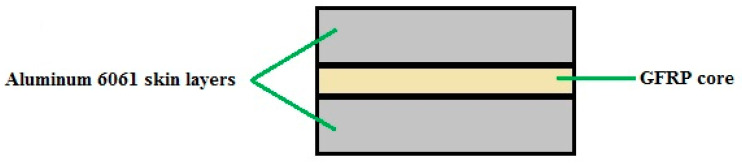
A schematic view of FML with skins and core.

In this study, for fabrication of FMLs, the aluminum alloy 6061-T6 with a length of 120 mm, width of 15 mm and thickness of 1 mm was used as the FML skin and the unidirectional E-glass fiber-reinforced polypropylene (PP) with a length of 120 mm, width of 15 mm and thickness of 0.3 mm was applied as the FML core materials. Also, the fiber volume fraction of the composites layer was approximately 40%. Since this paper examines the effect of the number of GFRP layers, FMLs with 2, 4 and 8 GFRP core layers are made and their CAF processes are investigated. A schematic view of FML with skins and core is shown in Figure 1. The glass fiber-reinforced prepreg was placed between the aluminum skins along the longitudinal direction of the sandwich beam. In order to ensure of fabrication of FMLs without delamination and defects, the prepared specimens were hot pressed at a pressure of 10 bar and a temperature of 180 °C for 10 min. As mentioned in the present study, the effects of process temperature and time, and also the effects of FML thickness and stacking sequences, were investigated.

As mentioned above, in order to investigate the effects of the number of layers as well as the type of stacking sequence on the springback behavior of FML in the creep age forming process, the stacking sequences presented in Table 1 were manufactured and tested based on the procedure presented in the following section. It should be noted that from experimental point of view, there are several types of conventional fiber-metal laminates that are manufactured with layers, such as 0, 90 and 45 composite layers. In this study, the main goal was to change the core stacking sequences using these mentioned layers, in such a way that the total bending modulus did not significantly change. Also, the mechanical properties of aluminum alloy and the composite material are presented in Table 2. It should be noted that the fabricated fiber–metal laminates had a width of 15 mm and a total length of 120 mm. Also, the data provided in Table 2 were determined from tensile and shear tests according to ASTM D3039, ASTM D3518 and ASTM E8.

**Figure 2:**
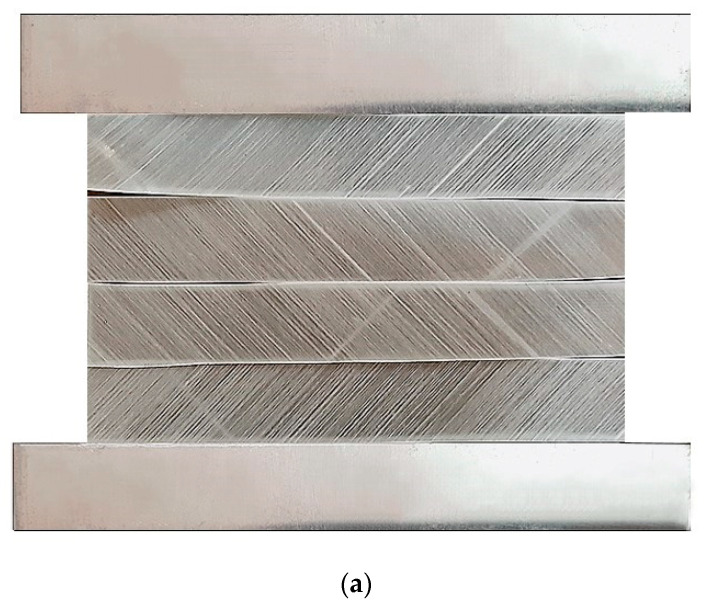

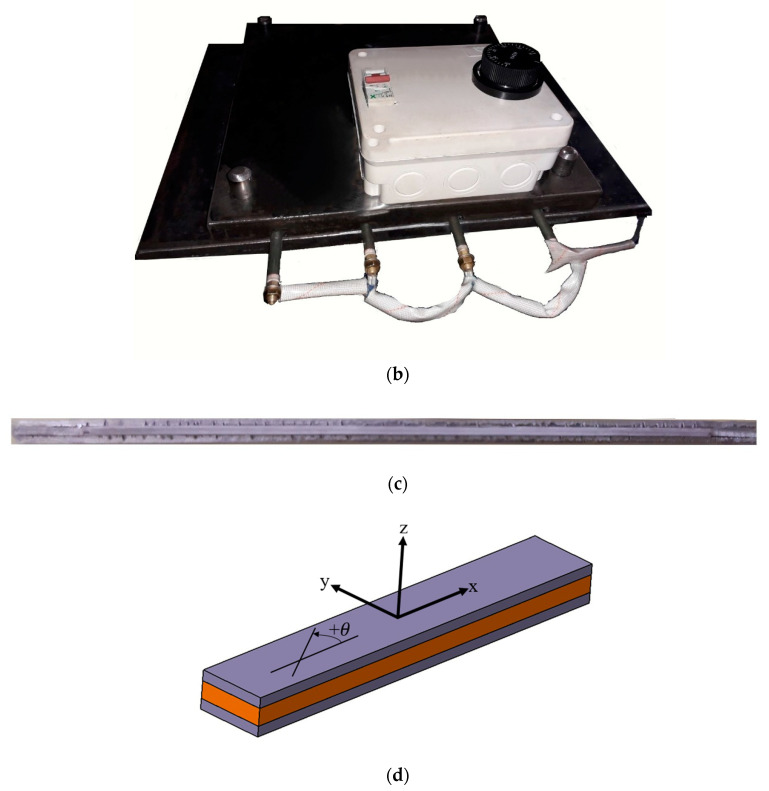
Preparation of FML samples; (**a**) Aluminum and GFRP layers, (**b**) Hot pressing process, (**c**) Prepared FML, (**d**) A schematic of GLARE specimen.

Figure 2 shows the steps for preparing the FML samples. The FML shown in Figure 2 is of type GLARE-5 and the same process was followed to prepare other FMLs.

### 2.2. CAF Process of FMLs

**Figure 3:**
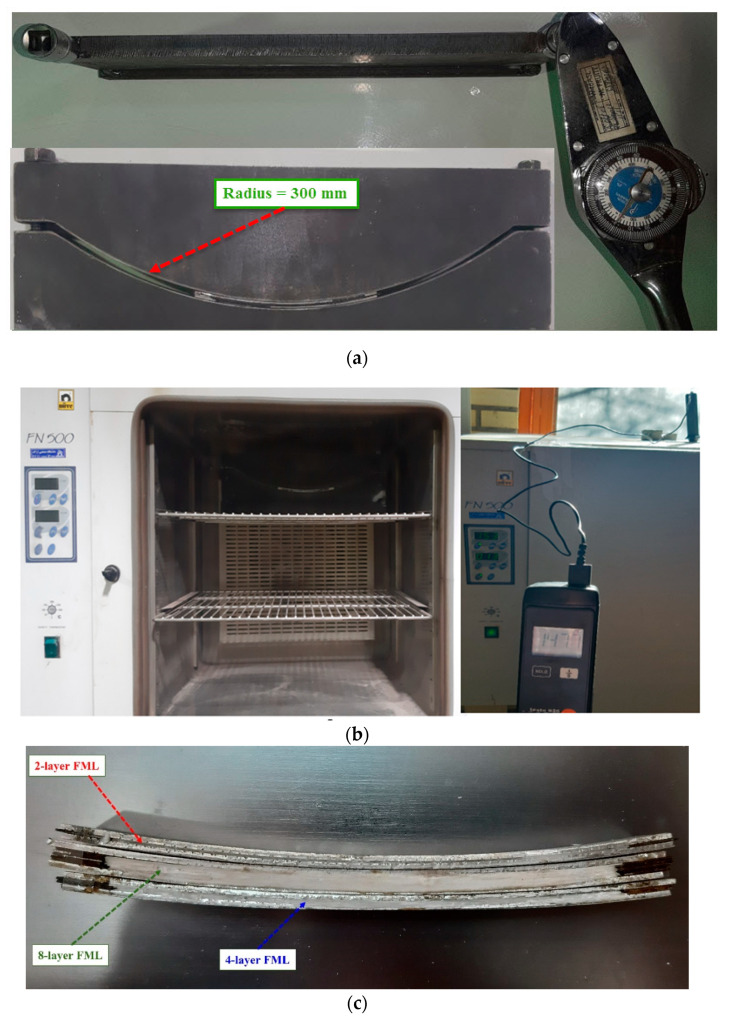
(**a**) A FML that is placed between die components with torque meter, (**b**) The entire assembly in the furnace, (**c**) Some creep age formed FMLs.

For the CAF process, a die set containing parts with circular surfaces with radiuses of 300 mm was employed. The CAF experiments were performed in a furnace with an accuracy of ±2 °C for the adjusted temperatures, to provide the desired temperature. The prepared FMLs were constrained between the die components with the bolts on each die half. Also, using a torque meter, the same loads were applied to the bolts, which created symmetrical condition in the CAF of FMLs. However, the entire die components and FML were placed in the furnace, and isothermal heat was applied to them. After aging the FMLs in the furnace based on adjusted temperatures and times, the entire assembly was cooled slowly in the furnace. In Figure 3a, a FML that was placed between die components with torque meter has been shown. Also, in Figure 3b, the entire assembly in the furnace is seen. In Figure 3c, some creep age formed FMLs show that the CAF can successfully form the FMLs. It is worth mentioning that after manufacturing the samples and performing the test, each of the samples was visually inspected to check for defects and delamination. Due to the narrow width of the beam, any delamination at the interface could be visually observed.

### 2.3. Springback Measurement

**Figure 4:**
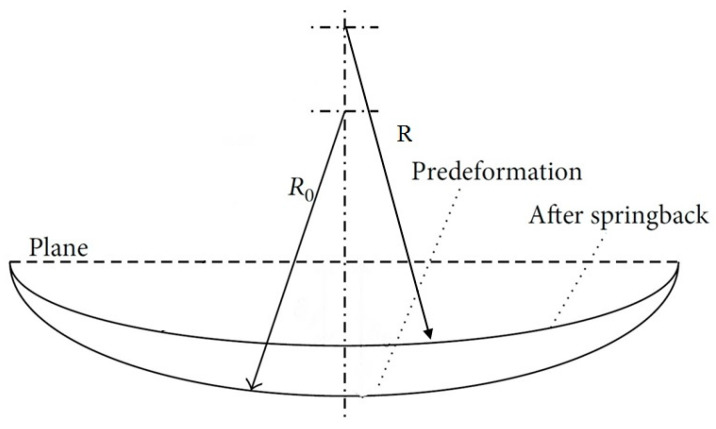
Schematic view of a creep age formed FML before and after the springback phenomenon. (Adapted from [27], with permission from Springer Nature, 2021).

After the CAF, the springback of the formed FMLs was measured by an Easson ENC-565 coordinate measuring machine (CMM). The accuracy of measurements for this device is 0.5 μm. The amount of springback indicates the dimensional accuracy of the formed FMLs and is one of the most important features in the forming processes. As shown in Figure 4, the springback can be calculated on a creep age formed FML before and after forming. The equation for calculating the springback is presented as Equation (1). The springback of the formed FMLs was measured by an Easson ENC-565 coordinate measuring machine (CMM). The accuracy of measurements for this device was 0.5 μm.

(1) $$\mathrm{Springback}\left(\%\right)=\left(\left(R/R_{0}\right)-1\right)\times 100$$

## 3. Results and Discussion

**Figure 5:**
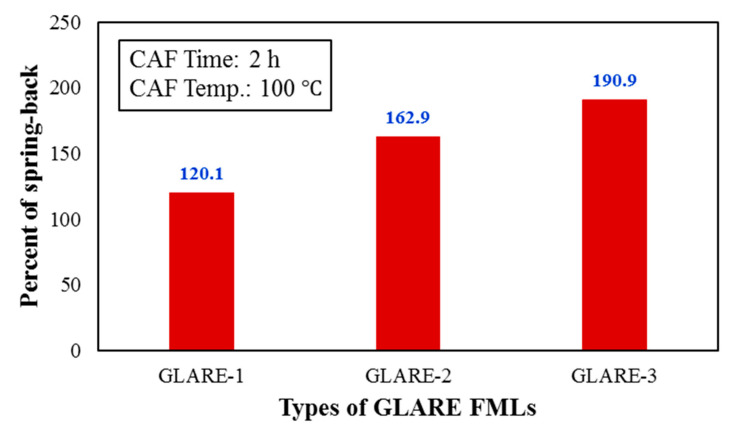
Effect of thickness of composites layer on the springback behavior of GLARE FMLs at CAF time 2 h and temperature 100 °C.

**Figure 6:**
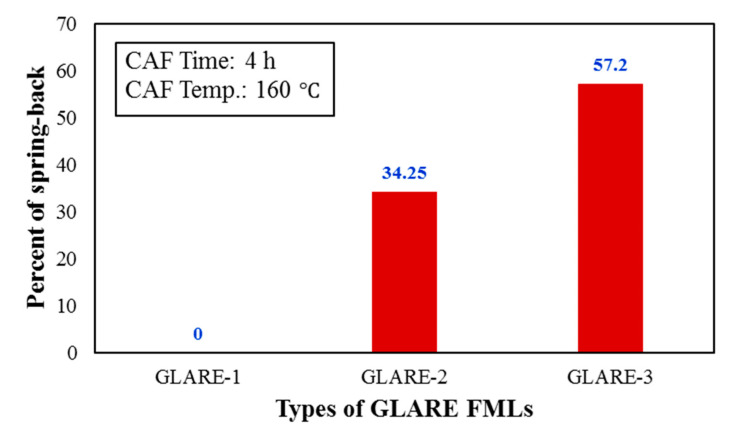
Effect of thickness of composites layer on the springback behavior of GLARE FMLs at CAF time 4 h and temperature 160 °C.

First, the simultaneous effect of temperature and creep time on the amount of FML springback is investigated and the experimental results are illustrated in Figure 5 and Figure 6. The results show that by increasing temperature and time, the amount of springback has decreased significantly. This is due to the diffusive nature of creep directly under the influence of temperature and time. Therefore, as the time and temperature increase and elastic strains decreases, the creep strains increase and hence, the springback decreases.

**Figure 7:**
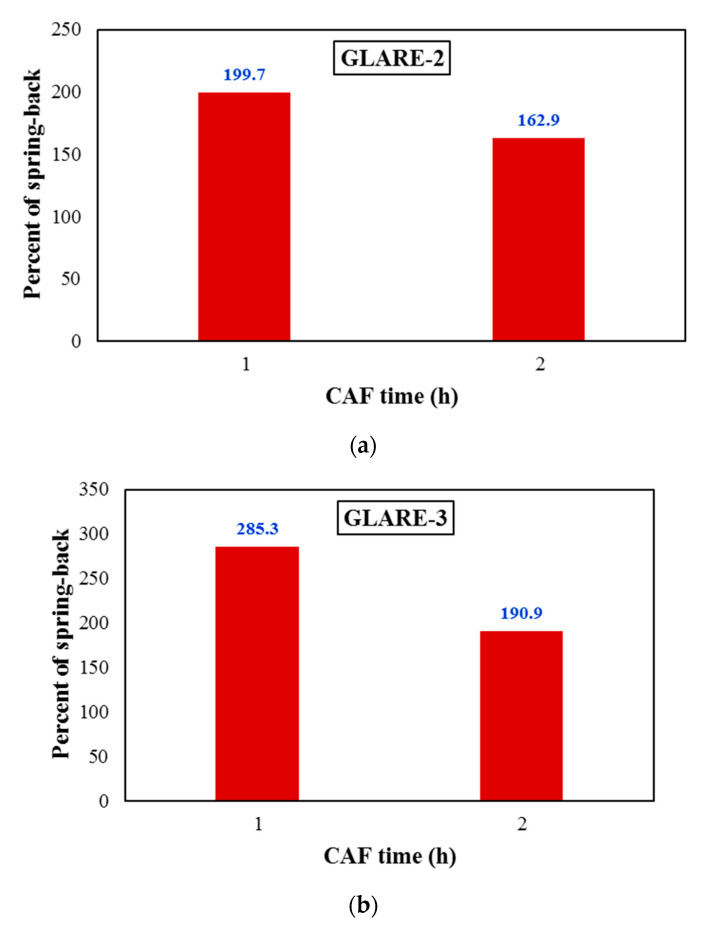
Effect of CAF time on the springback behavior of (**a**) GLARE-2 and (**b**) GLARE-3 at CAF temperature 100 °C.

In addition, the simultaneous effect of these two parameters on low-thickness FMLs is more than higher-thickness ones. In addition, it can be found that by increasing the thickness of the FMLs at each temperature and time, the amount of springback increased. Figure 7 provides the effect of time on the springback behavior of GLARE-2 and GLARE-3 at CAF temperature 100 °C. The results exhibit that in both types of FMLs, the amount of springback decreases with increasing time. By comparing the results obtained in Figure 7a,b with those in Figure 5 and Figure 6, it can be seen that the effect of the temperature parameter on the amount of springback is greater than the effect of time because, as shown in the results of Figure 7, by doubling the creep time, the amount of springback decreased by a maximum of 33%. In order to better demonstrate this phenomenon, the experimental data is provided in Table 3.

The provided results in Table 3 show that by doubling the time, the springback of the specimens reduced by about 38%. In addition, it can be concluded that with a 60% increase in creep temperature, springback of the GLARE is diminished. So, it can be seen that the effect of temperature on the amount of springback is greater than the effect of time.

**Figure 8:**
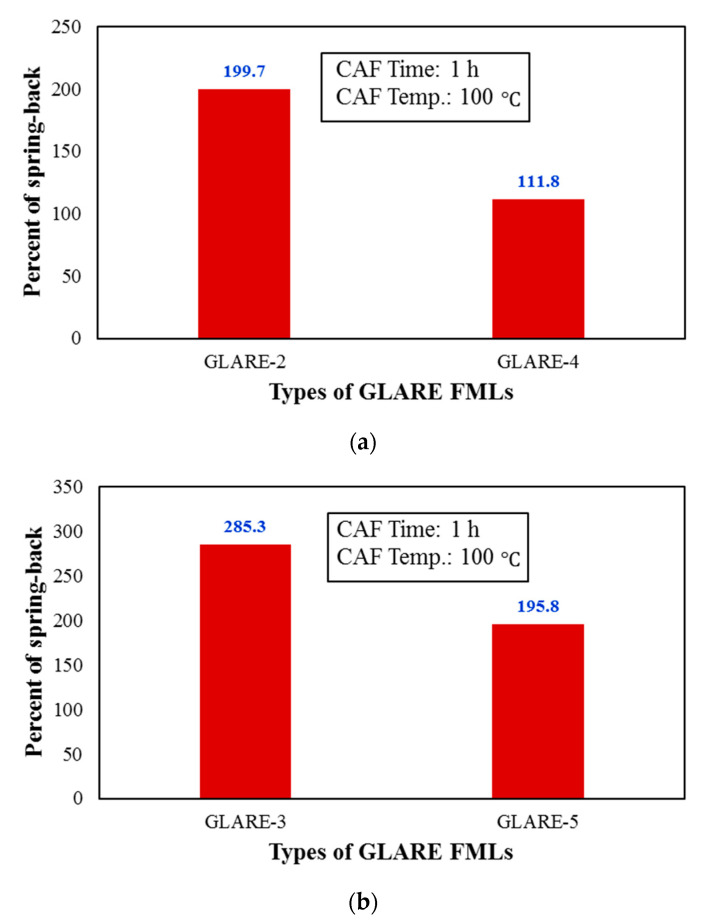
Effect of stacking sequence of composites layer on the springback behavior of (**a**) GLARE-2 and GLARE-4 (**b**) GLARE-3 and GLARE-5 FMLs.

Figure 8 shows the experimental results of the stacking sequence on the amount of springback for two types of FMLs with different thicknesses. As shown in this figure, the use of ±45° plies instead 0° ones significantly reduces the amount of springback.

Table 3 provides the amount of longitudinal flexural modulus, $E_{x}^{f}$, of each of the stacking sequences. The flexural modulus is also calculated using the following equation:

(2) $$E_{x}^{f}=\frac{12}{d_{11}h^{3}},\;\;\left[d\right]={\left(\left[D\right]-\left[B\right]{\left[A\right]}^{-1}\left[B\right]\right)}^{-1}$$

where, [A], [B] and [D] are the in-plane stiffness, bending-stretching coupling and bending stiffness matrices, respectively. The calculation of these matrices is provided in Ref. [29]. Also, $d_{11}$ is the array (1, 1) of the [d] matrix and h is the total thickness of the fiber–metal laminates. The mechanical properties of aluminum alloy and thermoplastic composites are provided in Table 4.

**Figure 9:**
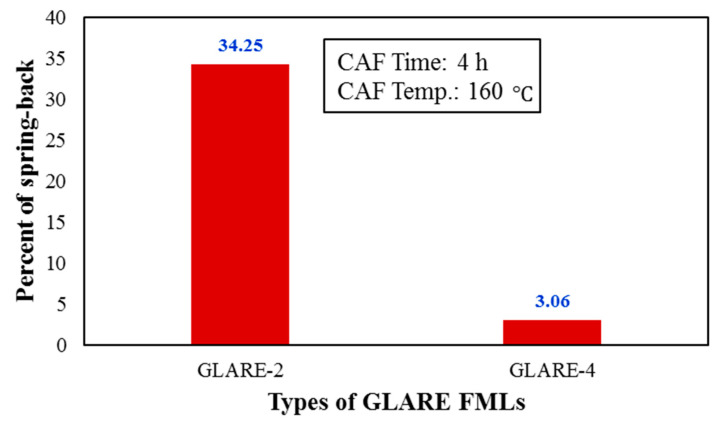
Effect of stacking sequence of composites layer on the springback behavior of GLARE-2 and GLARE-4 at CAF time 4 h and temperature 160 °C.

As an important result, it can be noted that the use of ±45 plies instead of 0° ones, with little change in the flexural modulus of FMLs (maximum less than 3.1% for GLARE-5), has been able to decrease about 42% of the springback. Hence, this issue can be considered a practical recommendation in manufacturing FML for CAF of these laminates, to reduce the effects of springback. Figure 9 shows the effect of the stacking sequence on the springback behavior of CAF of FMLs at another temperature and time. The presented results show a similar reduction trend in amount of springback.

## 4. Conclusions

In this paper, the creep age forming process of fiber metal laminates was experimentally studied. The FMLs were fabricated with aluminum alloy 6061 as the skin and E-glass fiber-reinforced polypropylene (PP) as the core. Hot pressing at a pressure of 10 bar and temperature of 180 °C for 10 min was performed to ensure the fabrication of FMLs without delamination and defects. The effects of CAF time and temperature and thickness and stacking sequences of composite layers in the FML core on the amount of springback were estimated. For the CAF process, the FMLs were constrained between die components and the die and FML were placed in a furnace. The isothermal heat was applied in the furnace for the CAF process. After aging, the die and FML were cooled slowly in the furnace. Then, the springback of creep age formed FMLs was calculated. The experimental results indicate that CAF time and temperature, as well as thickness and stacking sequences of composite layers, have significant effects on the springback behavior of GLARE FMLs. In addition, it can be found that the temperature affects the springback of FMLs more than the CAF time. As a significant result, it is worth noting that usage of ±45° plies instead of 0° angle layers as the FML core can reduce the springback of thick FMLs by more than 42%.

## Figures and Tables

**Table 1:** Manufactured FMLs with different stacking sequences.

Types	Stacking Sequence	Total Thickness (mm)
GLARE-1	${\left[Al/0\right]}_{s}$	2.4
GLARE-2	${\left[Al/0_{2}\right]}_{s}$	3.2
GLARE-3	${\left[Al/0_{4}\right]}_{s}$	4.4
GLARE-4	${\left[Al/\pm 45\right]}_{s}$	3.2
GLARE-5	${\left[Al/\pm 45/\mp 45\right]}_{s}$	4.4

**Table 2:** Mechanical properties of E-glass fiber-reinforced polypropylene (PP) and Aluminum alloy 6061.

E-Glass Fiber Reinforced Polypropylene (PP)			
$E_{1}\;\left(\mathrm{GPa}\right)$	$E_{2}\;\left(\mathrm{GPa}\right)$	$G_{12}\;\left(\mathrm{GPa}\right)$	$\nu_{12}$
23	6.8	3.8	0.35
**Aluminum alloy 6061**			
E (GPa)		ν	
70.2		0.33	

**Table 3:** Effect of time and temperature on the springback of GLARE-1.

Sample	Time (h)	Temperature (°C)	Springback (%)
1	2	100	120.1
2	4	100	74.4
3	4	160	0

**Table 4:** The longitudinal flexural modulus, $E_{x}^{f}\;\left(\mathrm{GPa}\right)$, of GLARE FMLs with different stacking sequence of composite materials.

GLARE-2	GLARE-3	GLARE-4	GLARE-5
67.71	62.5	67.06	60.55
